# Investigation of the relationship between fluconazole susceptibility, proteinase activity and *ERG11-SAP2* Expression in *Candida albicans* strains isolated from clinical samples

**DOI:** 10.22034/cmm.2024.345311.1585

**Published:** 2024-12-31

**Authors:** Nagihan Ege, Şükrü Öksüz, Emel Akbaş, Emel Çalişkan, Mehmet Ali Sungur

**Affiliations:** 1 Duzce Ataturk State Hospital, Deparment of Medical Microbiology, Duzce, Turkey; 2 Duzce University, Medical Faculty, Deparment of Medical Microbiology, Duzce, Turkey; 3 Duzce University, Medical Faculty, Deparment of Biostatistics and Medical Informatics, Duzce, Turkey

**Keywords:** *Candida albicans*, Fluconazole, Proteinase, Tolerance

## Abstract

**Background and Purpose:**

*Candida albicans* is currently recognised as an opportunistic pathogen that can cause many invasive infections. Resistance mechanisms and fungal virulence factors play an important role in the effectiveness of treatment. The aim of this study was to investigate the relationship between fluconazole resistance, proteinase activity and *ERG11* (sterol 14-demethylase)- *SAP2* (secreted aspartic protease 2) gene expression levels in *C.albicans* strains.

**Materials and Methods:**

*Candida albicans* strains isolated from patient samples sent to Medical Microbiology laboratory of Düzce University from various clinics were included in the study. Fluconazole susceptibilities of the isolates were determined by broth microdilution method. The increase in fluconazole MIC values at 48 hours and proteinase activities of the isolates were analysed. *ERG11* and *SAP2* gene expression levels were measured by real time qPCR.

**Results:**

Fluconazole resistance rate was found to be 3.14% in 127 *C. albicans* strains. A moderate positive correlation was found between *ERG11* and *SAP2* values (p=0.029, r:0.655, p<0.001). There was no correlation between *SAP2/ERG11* expression levels and fluconazole resistance. Proteinase positivity was detected in 81.1%, of 127 strains and no statistically significant correlation was found between proteinase activities and *SAP2/ERG11* expression levels. While there was a statistically significant relationship between *ERG11* expression levels and 48th hour MIC elevation, there was no statistically significant relationship between *SAP2* levels and 48th hour MIC elevation.

**Conclusion:**

In addition to the moderate positive correlation between *ERG11* and *SAP2* values, a significant correlation was found between *ERG11* expression and fluconazole tolerance.

## Introduction

The most common invasive systemic mycosis agents in the world are *Candida* species, especially *C.albicans* [ 1 ]. *C.albicans* can survive in many different environmental conditions. Due to this ability, it can proliferate in many regions of the human body and may cause various diseases ranging from superficial infections to life-threatening serious infections, especially in immunocompromised patients [ 2 , 3 ].

Treatment of *Candida* infections usually requires long-term drug use. Azole antifungals are primarily preferred for treatment. As a result of long-term and repeated antifungal use, resistant *Candida* strains are encountered. When the studies conducted in the last 20 years on fluconazole susceptibility of *C. albicans* are analysed, it is seen that the resistance rate is gradually increasing [ 4 ]. In a study conducted in different European countries, it was found out that resistance to azole antifungal drugs and other antifungals develop over time [ 5 ]. Accordingly, antifungal resistance is likely to become an important public health problem in the future. Many mechanisms responsible for azole resistance in *Candida* strains have been suggested, and it has been determined that more than one mechanism play a significant role in fluconazole resistance. Among these mechanisms, modification in the lanosterol demethylase (*ERG11*p) and ergosterol biosynthesis is the most important. Another important mechanisms is the change in lanosterol demethylase (*ERG11*p) and ergosterol biosynthesis [ 6 ]. Other important factors include mutations in UPC2 (Zn2-Cys6 transcription factor uptake control) transcriptıon genes such as tac1 and mrr1 and overexpression of efflux pumps linked to CDR1, CDR2, MDR1, PDR16 and SNQ2 transporter genes [ 7 ].There are many virulence factors in *Candida* species. These factors may play a role in different body regions at different stages of infection. Some of the known virulence factors are adhesion, yeast-hyphae dimorphism, phenotypic transformation, biofilm formation, hydrolytic enzymes, survival at normal or febrile body temperature (37°-39°C), and rapid adaptation to microchanges in the host [ 8 ]. Although recent publications have shown that proteinase, one of the virulence factors of *C. albicans*, may be associated with fluconazole resistance, the relationship between the expression of *SAP2*, one of the most important proteinase genes, and drug resistance remains unclear [ 9 ]. The aim of this study was to investigate the fluconazole susceptibility and tolerance of *C. albicans* from clinical samples and their relationship with the expression levels of *ERG11/ SAP2* genes and proteinase activity.

## Materials and Methods

*C. albicans* strains isolated from various clinical specimens sent to the Medical Microbiology Laboratory of Düzce University Medical Faculty between 01.08.2021 and 01.08.2022 were included in this study. In case of more than one sample from the same patient, the strains isolated from the first sample were used. Furthermore, an ethical clearance was obtained from Düzce University Non-Interventional Health Research Ethics Committee with decision number 2022/133 on 04.07.2022 prior to the conduct of the study. 

### Identification of Candida albicans strains

The patient samples were inoculated on Sabouraud Dextrose agar (SDA) (Condalab, Spain) medium, then placed in Eppendorf tubes containing brain-heart infusion broth (Plasmatec, UK). After 24 hours of incubation, the media were stored at -20°C until the commencement of the study. On the first day of the study, the Eppendorf tubes were kept at 37°C for 2 hours and inoculated on SDA medium and incubated at the same temperature for 18-24 hours. After the incubation period, the strains were identified using germ tube test, microscopic evaluation on corn flour-tween 80 agar by Dalmau plate method, growth characteristics on chromogenic medium, and Vitek 2 (bioMerieux, France) automated system were used [ 2 , 10 ].

### Determination of proteinase activity

Detection of proteinase activity was done using the enzyme activity of SAP by the yeast nitrogen base-bovine serum albumin YNB–BSA agar plate method [ 11 ]. Proteinase activity was tested using two different media. The first medium used was 1% bovine serum albumin (BSA) (Sigma-Aldrich, USA). For this purpose, a medium containing 2 g of BSA, 1.45 g of YNB (Difco Laboratories, Detroit, MI, USA), 20 g of glucose and 20 g of agar in one liter of distilled water was prepared [ 11 , 12 ]. Agar and glucose were mixed in 900 ml of distilled water and sterilized in an autoclave. Afterwhich, BSA and YNB were dissolved in 100 ml of distilled water and passed through a 0.22 μm paper filter (Isolab, Turkey). It was added to the first mixture, which was removed from the autoclave and cooled at 50 °C. For the second media, the same medium was used. However, 1 g KH_2_PO_4_ and 0.5 g MgSO_4_.7 H_2_O were added used in addition to the components of the first medium. Afterwhich, the medium was sterilized using an autoclave. The sterile media were poured into 9 cm petri dishes and stored at 4 °C. Additinally, a clinical isolate of *C. albicans* with known antibiogram profile (C. albicans ATCC 90028 strain) was used as positive control for the isolates.

Stock cultures for the yeast strains to be tested were inoculated on SDA medium. They were incubated at 37 °C for 24 hours. Pure colonies were taken using a sterile a loop and suspended on sterile distilled water. Afterwhich, the solution was standardized using 0.5 McFarland. 10 microliters of this suspension were taken with a micropipette and placed in a medium with a maximum of 4 strains, and the medium was incubated at 37 °C for 1 week. The diameters of the zone of inhibition at the end of the incubation period and the transparent zone formed around it due to protein degradation were measured. Proteinase activity was determined by the ratio of the sum of the colony diameter and the transparent zone diameter formed around the colony to the colony diameter and expressed as the Prz value. The Prz value was evaluated as negative if it was 1.0; 1+ if it was 0.9-1; 2+ if it was 0.89-0.8; 3+ if it was 0.79-0.7; and 4+ if it was ≤ 0.69 [ 13 ].

### Antifungal susceptibility test for fluconazole

Antifungal susceptibilities of the strains included in the study were analysed and interpreted according to the recommendations of EUCAST E.DEF 7.3.2 guideline, which is one of the reference methods in broth microdilution method. For fluconazole, MIC values of ≥ 8 µg/ml was considered as resistant (R), 4 µg/ml as dose-dependent susceptible (DDS) and ≤ 2 µg/ml as susceptible (S) [ 14 ]. On the, the 24^th^ hour of incubation, the MIC values of the strains were recorded. Afterwhich, the incubation period was extended to 48 hours in order to evaluate their tolerance and re-evaluation was performed at the end of 48 hours. 

### Quantitative Reverse Transcriptase Polymerase Chain Reaction (qRT-PZT)

Hybrid-R^TM^ (GeneAll®, Korea) kit was used for RNA isolation from the strains in accordance with the manufacturer's recommendations. After RNA isolation, cDNA synthesis phase was started. cDNA Synthesis Kit (A.B.T.^TM^, Turkey) was used for cDNA synthesis. After obtaining cDNA, real-time qPCR was performed. For this, 2X qPCR SYBR-Green MasterMix (A.B.T.^TM^, Turkey) kit was used. ACT1 was used as the housekeeping gene and *Candida albicans* ATCC 90028 was used as the reference strain. The primer sequences used in this study are shown in Table 1. 

**Table 1:** Primer sequences used in real time qPCR

Primer name	Primer sequence (5'-3')
*ERG11*-F	ACTCATGGGGTTGCCAATGTT
*ERG11*-R	GAGCAGCATCACGTCTCCAA
ACT1-F	AGGTTTGGAAGCTGCTGGTA
ACT1-R	ACGTTCAGCAATACCTGGGA
*SAP2*-F	AACAACAACCCACTAGACATCACC
*SAP2*-R	TGACCATTAGTAACTGGGAATGCTTTAGGA

### Relative quantitation calculation

Relative expressions were calculated by the ΔΔCT method. ACT1 gene was used as a reference and *C. albicans* ATCC 90028 strain was used for normalization.

### Statistical Analyses

Statistical evaluation of the data was done with IBM SPSS v.22 (IBM Corp. Released 2013. IBM SPSS Statistics for Windows, Version 22.0. Armonk, NY: IBM Corp.) package program. The distribution of numerical data was examined with the Kolmogorov-Smirnov test, the Mann-Whitney U test was used in group comparisons, and the analysis of categorical data was done with the Fisher-Freeman-Halton test. Spearman’s rho was used for correlation analysis. Descriptive statistics of numerical data are presented as median value, minimum-maximum, and categorical data are presented as numbers and percentages, and 0.5 level of significance was used.

## Results

A total of 127 *C. albicans* strains were evaluated in this study. 79 (62%) of the samples belonged to female and 48 (38%) to male patients. The age range of the patients was 0-93 years with a mean age of 55.8 years and a median value of 63 years. In this study, 56 (45%) yeasts were isolated from inpatient wards, 47 (37%) from outpatient clinics and 24 (18%) from intensive care units. In this study, comparison of *Candida* strains from both outpatients or inpatients was not considered. The distribution of the studied yeasts according to sample type is shown in Table 2.

**Table 2:** Distribution of strains according to sample type

Sample type	Number	%	p
Urine	84	66.1	p<0.001
Vagina	17	13.3	
Sputum	12	9.4	
Blood	3	2.3	
Wound	3	2.3	
DTA*	3	2.3	
Blood catheter	1	0.78	
BAL**	1	0.78	
Peritoneum	1	0.78	
Pleura	1	0.78	
Abscess	1	0.78	
Total	127	100	

* Deep Tracheal Aspirate,

** Bronchoalveolar Lavage

A broth microdilution antifungal susceptibility test was performed on 127 isolated *C. albicans* strains. The evaluation performed with spectrophotometric microplate reader at 24 hours showed that 123 (96.86%) of the strains were susceptible to fluconazole while four strains (3.14%) were resistant. The distribution of the strains according to MIC values is presented given in Table 3. Accordingly, 38 (29.92%) of 127 strains showed an increase in MIC values, while 89 (70.08%) showed no change in MIC values. There were 3 strains that switched from susceptible profile to dose-dependent susceptible profile after MIC values increased. In two of the fluconazole resistant strains, MIC values increased by one dilution, while no change was observed in the others. 

**Table 3:** Distribution of strains according to fluconazole MIC values

MIC value	Number (n=127)		%
	n	%	
0.125	63	49.60	*96.86
0.25	46	36.22	
0.5	7	5.51	
1	4	3.14	
2	3	2.36	
16	3	2.36	**3.14
32	1	0.78	

* Ratio of susceptible strains,

** Ratio of resistant strains

Proteinase positivity was detected in 103 (81.11%) of 127 strains included in the study. The classification of the strains according to the level of proteinase activity is shown in Table 4. Proteinase levels of the strains included in the study were compared with fluconazole susceptibility values, but no statistically significant relationship was found between them (p=0.718). The comparison of proteinase levels of the strains according to their susceptibility to fluconazole is shown in Table 5. 

**Table 4:** Distribution of strains according to proteinase activity

Proteinase activity	Number (n)	%
4+	91	71.65
3+	11	8.66
2+	0	0
1+	1	0.78
Negative	24	18.89

**Table 5:** Relationship between fluconazole susceptibility of strains and proteinase activities (n)

Proteinase value	Fluconazole susceptibility		Total	p
	S	R		
No	24	0	24	0.718
1+	1	0	1	
3+	11	0	11	
4+	87	4	91	

*ERG11* and *SAP2* gene expression levels of 44 strains randomly selected from 127 strains were investigated by qPCR. When the *ERG11* expression levels of 44 strains were analysed, it was observed that 25 strains expressed more *ERG11* and 14 strains expressed less *ERG11* compared to ATCC strains. *ERG11* expression was not observed in five strains. When the *SAP2* expression levels of 44 PCR strains were analysed, it was found that 13 strains expressed more *SAP2* and 12 strains expressed less *SAP2* compared to the ATCC strain, while no *SAP2* expression was observed in 19 strains. The fold changes of the strains with higher expression levels compared to ATCC are shown in Table 6. According to PCR results a moderate positive correlation was detected between *ERG11* and *SAP2* expression levels in a total of 11 strains that expressed both *ERG11* and *SAP2* more than the ATCC strain and in a total of 26 strains that showed both *ERG11* and *SAP2* positivity. (p=0.029, r:0.655, p<0.001). When the relationship between *ERG11*, *SAP2* expression levels of the strains and fluconazole susceptibility was examined, no statistically significant relationship was found between them (Table 7). There was a significant correlation between fluconazole MIC elevation at 48. hours and *ERG11* expression levels (p=0.037), while no significant correlation was found between fluconazole MIC elevation and *SAP2* levels at the end of the same period. There was no significant difference between *ERG11* and *SAP2* expression in a total of 44 strains for which PCR could be performed, 19 of which produced no proteinase and 25 produced 4+ proteinase (Table 8).

**Table 6:** *ERG11* and *SAP2* expression levels of strains with high expression levels according to *Candida albicans* ATCC 90028

Strain number	*ERG11* floor change	SAP 2 fold change
6	2.4	-
16	1.19	1.3
29	333.15	168.9
45	-	1.04
63	1.06	-
67	2.05	4.4
94	3.42	-
128	4.41	4.23
206	2.59	2.38
325	5	-
384	1.91	1.35
391	2.21	-
416	2.7	-
464	67.65	-
483	3.79	-
486	1.34	1.39
496	5.98	2.8
546	10.71	2.08
577	1.04	-
613	2.48	46.21
629	11.96	-
633	1.25	-
635	-	1.43
638	1.01	-
645	4.09	-
712	72.01	12.82
721	15.14	-

**Table 7:** Comparison of fluconazole susceptibilities in relation to *ERG11* and *SAP2* expression

Expression type	Expression status (n)	Fluconazole susceptibility		p
		S	R	
*ERG11* expression	Negative (5 )	5	0	>0.999
	Less than ATCC (14 )	13	1	
	More than ATCC (25)	22	3	
	Total (44)	40	4	
*SAP2* expression	Negative (16 )	15	1	0.600
	Less than ATCC (15)	14	1	
	More than ATCC (13)	11	2	
**Total**		**40**	**4**	

**Table 8:** Relationship of *SAP2* and *ERG11* expression status with the presence of proteinase according to ATCC strain

Expression type	Feature (n)	Presence of proteinase (n)		p
		No	4+	
*SAP2* expression	Negative (16)	9	7	0.409
	Less than ATCC (15)	5	10	
	More than ATCC (13)	5	8	
	Toplam (44)	19	25	
*ERG11* expression	Negative (5)	2	3	0.415
	Less than ATCC (14)	4	10	
	More than ATCC (15)	13	12	
	Toplam (44)	19	25	

## Discussion

Nowadays, due to the widespread use of broad-spectrum antibiotics and immunosuppressive drugs, prolonged neutropenia due to cytotoxic therapy, increased catheter use, and interventional procedures such as major cardiac and abdominal surgery, *Candida* species are at the forefront of opportunistic nosocomial infection agents [ 15 ]. The problem of antifungal resistance observed as a result of the increased use of antifungal drugs due to this increase in *Candida* infections has accelerated studies investigating the mechanisms of resistance to these drugs [ 16 ]. Azole antifungals are frequently used in the treatment of *C. albicans* infections. However, resistance to azoles may develop with long-term use. Wang et al. [ 17 ] reported fluconazole resistance rate in *C. albicans* strains as 4.16% in an antifungal resistance surveillance study conducted in China in 2022. In a study conducted in the USA with 621 *C. albicans* strains, the fluconazole resistance rate was found to be 0.5% [ 18 ], Tiryaki et al. [ 19 ] did not find any fluconazole-resistant *C. albicans* strains in a study on *Candida* strains isolated from various patient samples. Erdem et al. [ 20 ] found the fluconazole resistance rate in *C. albicans* strains to be 1.6% in a study on hospital infections in 2012. In this study, the fluconazole resistance rate in *C. albicans* strains was found to be 3.16%. The resistance of *C. albicans* to azoles in this study was found to be higher compared to the results of Wang et al. [ 17 ]. This may be due to the antifungal use policies of the institutions where the studies were conducted.

Azole antifungals work by targetıng 14-alpha demethylase enzyme, which is controlled by the *ERG11* gene in the ergosterol biosynthesis pathway in fungal cells. Ergosterol is an important component of the fungal cell membrane. Interruption of its synthesis leads to the accumulation of 14a-methyl sterols. This inhibits membrane stability, permeability, and the activity of membrane-bound enzymes. Overexpression of the *ERG11* gene is considered one of the common azole resistance mechanisms in *C. albicans* strains [ 21 , 22 ]. In a study conducted in 2022, Suchodolski et al. [ 22 ] reported that increased *ERG11* expression in *C. albicans* contributed to increased ergosterol production and increased drug resistance of strains. In a study conducted by Feng et al. [ 23 ] in 2016, they found that *ERG11* expression was significantly higher in fluconazole-resistant strains of *C. albicans* compared to susceptible strains and reported that resistance to azoles in *C. albicans* isolates may be associated with mutations in the *ERG11* gene. Franz et al. [ 21 ] found that increased *ERG11* expression levels were correlated with fluconazole resistance in their study with azole-resistant *C. albicans* strains. Some studies have shown that there is no significant relationship between *ERG11* overexpression and azole resistance [ 24 , 25 ]. For example, Park and Perlin [ 24 ] found the *ERG11* overexpression rate to be 12% in their study with 32 azole-resistant *C. albicans* strains and found no statistically significant correlation with azole resistance. White et al. [ 25 ] found no significant correlation between *ERG11* expression and fluconazole resistance in their study with equal numbers of fluconazole-sensitive and -resistant strains. They also stated that *ERG11* gene mutation is frequently seen in *C. albicans* strains and is not reliably associated with resistance, and that the mechanisms causing azole resistance phenotype may be diverse. In our study, when the relationship between the numerical values ​​of *ERG11* expression and fluconazole susceptibility of 25 of 44 *C. albicans* strains that were found to express more *ERG11* than ATCC strains was examined, no statistically significant difference was found between *ERG11* expression and fluconazole resistance. Considering the mutations mentioned in the literature and the existence of different resistance mechanisms, it was thought that further studies using a large number of resistant *Candida* strains could contribute more to the determination of the relationship between *ERG11* *SAP2*, an important virulence factor for *C. albicans*, can provide nutrients for its own growth by degrading macromolecular proteins on the mucosal surface and also increases the ability of *C. albicans* to adhere to and invade the host. In a study conducted by Gerges et al. [ 26 ] with 181 *C. albicans* strains, *SAP2* gene expression was detected in 69.6% of the strains by RT-qPCR analysis and a significant relationship was found between proteinase production and fluconazole resistance. In this study, it was observed that 13 (29.5%) of 44 *C. albicans* strains that were found to express *SAP2* more than ATCC strains had no significant relationship with fluconazole sensitivity. In addition, when the expression levels of 26 strains showing *ERG11* and *SAP2* positivity were examined, a moderate positive correlation was observed between *ERG11* and *SAP2* values, similar to the study of Feng et al. [ 9 ]. It is thought that *ERG11* and *SAP2* may have a certain relationship in the formation of antifungal resistance in *C. albicans* and may act together in the emergence of resistance to azole drugs. Since the number of azole-resistant strains in this study was low, no significant correlation was found between the numerical values of *SAP2* expression and fluconazole sensitivity of the strains that were found to express more *SAP2* than the control strains. 

Proteinases mediate very important biological activities such as hyphae production, adhesion and invasion in *Candida* infection [ 27 ]. In various studies, the presence of proteinase in *C. albicans* strains varies between 57-81% [ 13 , 26 , 28 - 30 ]. In this study, proteinase positivity was detected in 103 of 127 strains (81.11%). 71.65% of the strains were found to have strong, 8.66% moderate and 0.78% weak proteinase activity.

The ability of *C. albicans* to produce extracellular enzymes may contribute to its virulence. It can produce different enzymes, and their amount and potency vary depending on the site of isolation [ 31 ]. Garaghani et al. [ 32 ] detected high and moderate levels of proteinase activity in 97.1% of *C. albicans* strains isolated from vulvovaginal samples and found no significant difference in proteinase activity among genotypes [ 33 ]. Another study found that an aspartyl proteinase secreted by *C. albicans*, in addition to its protease activity, contains the RGD (RGDRGD) motif required for binding to host integrins and activates immune cells to induce proinflammatory cytokines. In this way, it has been reported that *C. albicans* can penetrate deeper into the oral mucosa and spread systemically by taking advantage of the disruption of intercellular connections [ 33 ].The presence of proteinase in this study was found to be consistent with the literature and no significant relationship was found between fluconazole sensitivity and proteinase presence. Studies examining the relationship between *ERG11* expression levels and proteinase activity of strains are limited. Said et al. [ 34 ] found a positive correlation between proteinase activity and *ERG11* expression levels in fluconazole-resistant *Candida* strains. However, no statistically significant relationship was found between the numerical values of proteinase activity of the strains and *ERG11* expression levels in this study (p=0.126). By increasing the number of resistant strains, different result may be obtained in future studies. 

Antifungal tolerance refers to the ability of fungal cells to survive in the presence of drug concentrations sufficient to inhibit cell growth, i.e., drug concentrations above the MIC breakpoint. Tolerance to azole agents is important because it limits the effectiveness of drug treatments [ 35 ]. Mashaly et al. [ 28 ] found a fluconazole tolerance rate of 33% in a study conducted in Egypt with 88 *C. albicans* isolates in 2022. In this study, no strain has been found to switch from a susceptible profile to a resistant profile among the isolated strains after 48 hours of extended incubation. However, three strains has been found to switched from a susceptible profile to a 'dose-dependent susceptible' profile. This results showed that fluconazole tolerance is low in the area where the study is conducted.

Moreover, the inability to distinguish the infectious agent or colonization of the strains included in the study, the inability to perform *ERG11* and *SAP2* PCR tests on all strains due to insufficient financial resources, and the small number of fluconazole-resistant strains were not considered in this study. 

## Conclusion

In conclusion, in addition to the moderate positive correlation between *ERG11* and *SAP2* values, a significant correlation was found between *ERG11* expression and fluconazole tolerance. Considering that the presence of proteinase is a virulence factor in *C. albicans* strains, these results support the idea that large scale studies are needed to determine the relationship between the SAP family and antifungal resistance as well as the relationship between *ERG11* and *SAP2* values.
